# Laser interstitial thermal therapy for IDH wild-type recurrent glioblastoma: a Scandinavian two-center cohort study

**DOI:** 10.1007/s00701-026-06771-0

**Published:** 2026-01-23

**Authors:** Silas H. Nielsen, Sara Tabari, Jane Skjøth-Rasmussen, Margret Jensdottir, Thomas Urup, Adam E. Hansen, Jiri Bartek, Rune Rasmussen

**Affiliations:** 1 Department of Neurosurgery, Rigshospitalet, Copenhagen University Hospital, Copenhagen, Denmark; 2 Danish Comprehensive Cancer Center Brain Tumor Center, Copenhagen, Denmark; 3 Department of Neurosurgery, Karolinska University Hospital, Stockholm, Sweden; 4 Department of Clinical Neuroscience, Karolinska Institute, Stockholm, Sweden; 5 Department of Clinical Medicine, University of Copenhagen, Copenhagen, Denmark; 6 Department of Oncology, Rigshospitalet, Copenhagen, Denmark; 7 Department of Radiology, Rigshospitalet, Copenhagen, Denmark

**Keywords:** Laser interstitial thermal therapy, Stereotactic laser ablation, Recurrent glioblastoma, Neuro-oncology, Neurosurgery

## Abstract

**Background:**

Management of recurrent glioblastoma (rGBM) remains challenging, particularly for deep-seated or eloquent recurrences not amenable to open surgery, and no standardized effective treatment exists for recurrent disease. European data on MR-guided laser interstitial thermal therapy (LITT) are scarce. This study presents the first Scandinavian experience with LITT for IDH-wild-type rGBM, within publicly funded healthcare systems providing population-wide access to care.

**Methods:**

Retrospective data from 30 consecutive patients with histologically confirmed IDH-wild-type rGBM treated with LITT at two Scandinavian centers between January 2019 and May 2024 were analyzed. Demographics, ECOG performance status, procedural parameters, adverse events, and survival outcomes were collected. Kaplan–Meier estimates were used for progression-free survival (PFS) and overall survival (OS).

**Results:**

Mean age was 57 years (SD 10.2); 83% had pre-LITT ECOG 0. Complete and ≥ 90% ablation of contrast enhancement was achieved in 83% and 97% of cases, respectively. Median hospital stay was 1 day, with 87% discharged by day 2. Adverse events occurred in 33%, predominantly transient neurological deficits (27%) and infections (7%); 10% had persistent deficits. Median OS after LITT was 13.5 months (95% CI 11.6–20.5) and median PFS was 4.3 months (95% CI 2.6–9.1); 37% remained progression-free at 6 months.

**Conclusions:**

LITT was feasible and well tolerated in this two-center Scandinavian cohort, with short hospitalization and low morbidity. Survival outcomes were within the range reported in previous international series. While these findings provide preliminary real-world data on LITT use within Scandinavian publicly funded healthcare systems, the limited sample size and retrospective design preclude definitive conclusions. Prospective, controlled studies are warranted to clarify the clinical role of LITT in recurrent glioblastoma.

## Introduction

Glioblastoma (GBM) is the most common primary malignant brain tumor in adults. Despite advances in surgical techniques, radiation therapy, and chemotherapy, prognosis remains poor [14]. Standard first-line treatment consists of maximal safe surgical resection followed by radiotherapy with concomitant and adjuvant temozolomide, a regimen commonly referred to as the Stupp protocol [25]. Even with optimal treatment, the median overall survival is approximately 15 to 18 months, and most patients experience tumor recurrence within the first year of diagnosis [1, 2, 17].

Management of recurrent GBM (rGBM) poses a significant clinical challenge. Therapeutic options include repeat surgical resection, re-irradiation, systemic chemotherapy, or enrollment in clinical trials. However, there is no universally accepted standard of care for recurrent disease. Repeat surgical resection can offer survival benefits in selected patients with favorable performance status and accessible tumors, but it is often limited by the tumor’s anatomical location, multifocality, or the patient's clinical condition [11, 19].

Laser interstitial thermal therapy (LITT) has emerged as a minimally invasive surgical alternative for cytoreductive treatment, particularly in deep-seated or eloquent brain regions where open surgery carries significant risk [12]. LITT uses an MRI-guided laser catheter to thermally ablate tumor tissue. Several retrospective studies and meta-analyses have suggested that LITT may offer similar survival outcomes to open resection, with median post-procedural survival ranging from 9 to 13 months and progression-free survival (PFS) typically reported around 4 to 6 months [15, 21]. Furthermore, LITT is associated with shorter hospital stays and potentially faster initiation of adjuvant therapies [4, 26]. The reported complication rate varies, with transient neurological deficits being the most common; however, the incidence of permanent morbidity remains relatively low at around 4% [6, 15].

Despite its increasing adoption, the evidence supporting LITT is primarily retrospective, with heterogeneity in patient selection, treatment protocols, and outcome reporting. Furthermore, most articles report data on mixed cohorts of high-grade gliomas with little or no data on biopsy verification. Data from European centers remain limited. This study aims to contribute to the growing body of literature by presenting the first two-center European experience with LITT in patients with rGBM.

We sought to evaluate the feasibility, safety, and efficacy of LITT as a treatment for rGBM in a two-center cohort, while identifying clinical and procedural factors associated with outcomes, in hopes of improving patient selection criteria in the future.

## Methods

### Inclusion

The study was approved by the National Danish Research Ethics Committee (Project ID: H-21047703) and the Swedish Ethical Review Authority (DNR 2017/1760–31 and 2025–03035-02). Patient data from both Rigshospitalet (*n* = 16) and Karolinska University Hospital (*n* = 14) were collected retrospectively. Eligible patients had histopathologically confirmed IDH-wildtype glioblastoma, had undergone prior surgical resection, and were treated with LITT for recurrent disease between January 1, 2019, and May 31, 2024. All cases were reviewed at a multidisciplinary tumor board. LITT was considered for patients with small, deep-seated recurrences in whom complete ablation coverage was anticipated. Non-invasive treatment options, such as re-irradiation with proton therapy or Gamma Knife radiosurgery were deemed unsuitable by the multidisciplinary board. Moreover, LITT was only considered for targets considered unsuitable for safe open resection or associated with a high anticipated risk of access-related morbidity. Patients who were appropriate candidates for standard repeat craniotomy were not selected for LITT. No additional exclusion criteria were applied.

### Data collection

Demographic, clinical, radiological, surgical, oncological and histopathological data were extracted from electronic medical records, radiological archives and the Visualase console. Demographics included patient age and sex. Preoperative parameters included prior surgical and oncological therapy, preoperative ECOG performance status (ECOG PS) and tumor characteristics including tumor volume, location, ventricular contact, laterality, multifocality, and distance from the resection cavity. Tumor and ablation volume segmentation was performed using Brainlab Elements software (Brainlab AG, Munich, Germany). Surgical parameters included number of lesions treated, ablations performed, number of laser catheters used, stereotactic technique (Leksell, Cosman-Robert-Wells, or Clearpoint SmartFrame Array), catheter replacement, diffusion tip length, biopsy result, ablation volume (as defined as the region inside the contrast-enhancing rim), and extent of ablation (as evaluated by the MRI description by a neuroradiologist and the treating physician). Postoperative parameters included adverse events, transient (resolved within 30 days) and persistent neurological deficits, postoperative ECOG PS, steroid use and hospital stay duration. Additional follow-up data included pattern of progression-free survival (PFS), PFS after 6 months, progression pattern (local vs. general), survival after LITT and survival after first resection.

### Surgical technique

All procedures were performed using the Medtronic Visualase™ MRI-guided laser ablation system.

At Karolinska University Hospital, the Leksell stereotactic frame system (Elekta Instruments AB, Stockholm, Sweden) was used for both biopsy and laser catheter placement. Intraoperative O-arm 3D Fluoroscopy provided image registration and confirmation of catheter position. The patient was then transferred to the clinical MRI suite, where a pre-ablation T1 sequence with a half-dose of contrast was acquired. Laser energy was delivered under real-time thermometry monitoring, and a postoperative T1 scan with half-dose contrast was obtained to assess ablation extent and to screen for any radiological complications.

At Rigshospitalet, the Cosman–Roberts–Wells (CRW) stereotactic frame system (Integra LifeSciences, Plainsboro, New Jersey, USA) was used to guide stereotactic biopsy and laser catheter placement. Registration was achieved with the AIRO mobile CT scanner, after which patients underwent ablation in the intraoperative MRI suite. Similar to Karolinska, a pre- and postoperative T1 with half-dose contrast was acquired. In one patient, biopsy and laser catheter placement were guided by the ClearPoint® SmartFrame Array MRI-guided stereotactic system (ClearPoint Neuro Inc., Solana Beach, California, USA) [16].

Biopsy was not routinely performed during the early phase of the programmes due to institutional caution regarding combining biopsy and LITT within the same operative session. With increasing experience and confidence in procedural workflow, both centers subsequently adopted routine biopsy prior to ablation.

### Statistics

Continuous variables were summarized as either mean ± standard deviation (SD) or median with interquartile range (IQR), depending on distributional normality assessed by visual inspection of histogram. Categorical variables are reported as counts and percentages. All baseline, tumor‐ and procedure‐related parameters (e.g. age, tumor volume, number of fibers, ablation coverage) were tabulated descriptively; no formal hypothesis testing was performed between centers or subgroups given the exploratory, single‐arm design.

Three time‐to‐event endpoints were analyzed: (1) progression‐free survival (PFS), defined as the time from the LITT treatment date to radiological progression based on the RANO criteria^11^ estimated by a neuroradiologist and the treating physician; (2) post-LITT overall survival (OS), defined as time from LITT to death or last follow-up; and (3) survival from primary surgical resection, defined from initial resection date to death or last follow-up. Survival functions were estimated by the Kaplan–Meier method and presented as median time (months) with 95% confidence intervals (CI). Patients alive or progression-free at last follow-up were censored at that date. All analyses were performed in R version 4.3.1 (R Foundation for Statistical Computing, Vienna, Austria) using the survival and survminer packages. A two-sided p-value < 0.05 was considered statistically significant for any exploratory subgroup comparisons or log-rank tests, although such comparisons were not a primary aim of this cohort study.

## Results

### Baseline characteristics

**Fig. 1:**
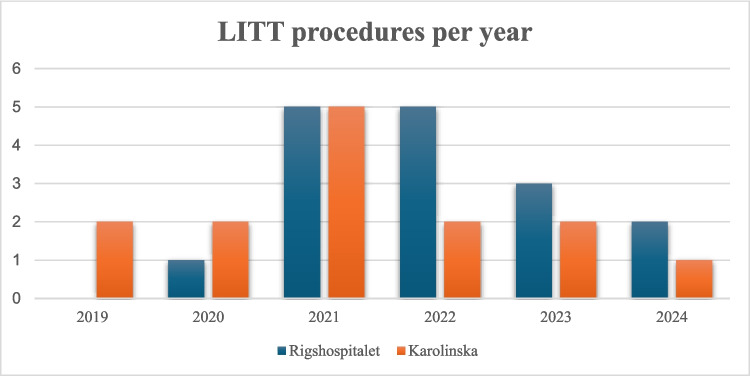
Annual number of LITT procedures for recurrent IDH-wildtype glioblastoma performed at Rigshospitalet and Karolinska University Hospital between 2019 and 2024

A total of 30 patients met the inclusion criteria: 14 treated at Karolinska University Hospital (Stockholm, Sweden) and 16 at Rigshospitalet (Copenhagen, Denmark) (Fig. 1, Table 1). The mean age at the time of LITT was 57.1 years (SD 10.2). There were 11 females (37%) and 19 males (63%). All tumors were histologically confirmed as glioblastoma (IDH-wildtype) and in 22 of 30 patients (73%) the MGMT promoter was methylated. Prior oncological therapy had been administered to all patients of whom 29 patients (97%) had received standard therapy according to the Stupp regimen. Twenty-three patients (77%) had undergone one prior surgical resection, six (20%) had two resections, and one patient (3%) had five prior resections. Pre-LITT Eastern Cooperative Oncology Group performance status (ECOG PS) was 0 in 25 patients (83%) and 1 in five patients (17%). Baseline characteristics were comparable between centers with respect to age, ECOG PS, tumor volume, and MGMT methylation status. No significant between-center differences were observed (all p > 0.1).

**Table 1:** Baseline characteristics

Characteristics	Value^1^
Age (yrs)	57.1 (10.2)
Sex	
Female	11/30 (37%)
Male	19/30 (63%)
Tumor classified as GBM	30/30 (100%)
IDH wildtype	30/30 (100%)
MGMT methylation	22/30 (73%)
Previous oncological therapy	30/30 (100%)
Previous radiotherapy	29/30 (97%)
Number of previous resections	
1	23/30 (77%)
2	6/30 (20%)
5	1/30 (3%)
Pre-LITT ECOG PS^2^	
0	25/30 (83%)
1	5/30 (17%)

^1^Mean (SD); n/total (%), ^2^ECOG Performance status

### Tumor characteristics

Tumor recurrence patterns and radiographic characteristics are detailed in Table 2. The temporal lobe was the most common site of recurrence (14/30; 47%), followed by the parietal (8/30; 27%), frontal (7/30; 23%), and basal ganglia (1/30; 3%). The median tumor volume at recurrence was 0.9 cm^3^ (IQR 0.4–1.6). Ventricular contact was observed in 13/30 (43%) and multifocal disease in 4/30 (13%). Laterality was right‐sided in 18/30 (60%) and left‐sided in 12/30 (40%). In terms of spatial relationship to the original resection cavity, 17/30 (57%) recurrences were within the cavity, 6/30 (20%) in the same lobe, 4/30 (13%) elsewhere in the same hemisphere, and 3/30 (10%) in the contralateral hemisphere. Finally, most recurrences were classified as local (< 2 cm) in 18/30 (60%), distant (> 2 cm) in 9/30 (30%), and both local and distant in 3/30 (10%).

**Table 2:** Tumor recurrence characteristics

Characteristics	Value^1^
**Tumor volume (cm**^**3**^**)**	0.9 (0.4, 1.6)
**Location**	
Basal ganglia	1/30 (3%)
Frontal	7/30 (23%)
Parietal	8/30 (27%)
Temporal	14/30 (47%)
**Ventricular contact**	13/30 (43%)
**Laterality**	
Left	12/30 (40%)
Right	18/30 (60%)
**Multifocality**	4/30 (13%)
**Recurrence location**	
In resection cavity	17/30 (57%)
Same lobe	6/30 (20%)
Same hemisphere	4/30 (13%)
Other hemisphere	3/30 (10%)
**Recurrence classification**	
Local (< 2 cm)	18/30 (60%)
Distant (> 2 cm)	9/30 (30%)
Local and distant	3/30 (10%)

^1^Median (IQR); n/total (%);

### LITT procedural parameters

Procedural details are summarized in Table 3. Most patients (27/30; 90%) underwent treatment of a single lesion, while three patients (10%) had two lesions ablated. The median number of ablations per patient was three (IQR 2–4), and the median number of fibers used was one (range 1–2); specifically, 24 patients (80%) were treated with a single fiber and six (20%) with two fibers. Regarding diffusion tip length, 28 patients (93%) were treated with the 10 mm tip and two (7%) with the 3 mm tip.

**Table 3:** LITT procedural parameters

Parameters	Value^1^
Number of lesions treated	
1	27/30 (90%)
2	3/30 (10%)
Number of fibers	
1	24/30 (80%)
2	6/30 (20%)
Length of diffusion tip	
3 mm	2/30 (7%)
10 mm	28/30 (93%)
Number of ablations	3 (2–4)
Stereotactic technique	
CRW	15/30 (50%)
Leksell	14/30 (47%)
Clearpoint Smartphrame	1/30 (3.3%)
Replacement of catheter	2/30 (6.7%)
Pre-LITT biopsy	19/30 (63%)
Biopsy diagnosis	
Tumor cells	18/19 (95%)
Reactive changes	1/19 (5%)
Lesion volume post-LITT (cm^3^)	2.4 (1.7, 4.5)
Extent of ablation	
60–90%	1/30 (3.3%)
90–95%	2/30 (6.7%)
95–99%	2/30 (6.7%)
100%	25/30 (83%)

^1^n/total (%); Median (IQR)

Laser catheter placement and any concurrent biopsy were performed using the following stereotactic systems: the Cosman–Roberts–Wells (CRW) frame in 15 patients (50%), the Leksell frame in 14 patients (47%), and Clearpoint Smartphrame Array in one patient (3%). Catheter replacement was required in 2/30 cases (7%) due to initial malposition. A biopsy at the time of LITT was undertaken in 19 patients (63%); of these, 18/19 (95%) confirmed viable tumor cells, and one (5%) showed reactive changes only.

The median post‐ablation lesion volume (within the contrast‐enhancing rim) was 2.4 cm^3^ (IQR 1.6–4.5). Ablation coverage of the contrast‐enhancing lesion was complete (100%) in 25/30 cases (83%), 95–99% in 2/30 (7%), 90–95% in 2/30 (7%), and 60–90% in 1/30 (3%).

### Procedural outcome and adverse advents

At first post‐LITT assessment, 19 of 30 patients (63%) maintained an ECOG PS of 0, ten patients (33%) had ECOG PS 1, and one (3%) had ECOG PS 3. A decline in ECOG PS occurred in 6 of 30 patients (20%), while 24 (80%) showed no change. Procedure-related adverse events were recorded in 10 of 30 patients (33%). Of those transient neurological deficits were documented in 8 patients (27%) and resolved within 30 days. Persisting neurological deficits beyond 30 days occurred in 3 patients (10%). Two patients had postoperative infection whereas one required local wound revision and one required open surgery for an intracranial abscess. Postoperative steroids were administered for a median of 7 days (IQR 6–13), including tapering. Initial dosing corresponded to a median prednisolone-equivalent dose of 100 mg/day. Several patients did not require postoperative steroids. All treated patients were tapered off steroids.

According to the Landriel Ibáñez classification, 80% of adverse events were Grade Ia –Ib (mild, requiring no or only simple medical treatment), 20% were Grade IIb (moderate, requiring surgical revision under general anesthesia), and no Grade III events occurred (Table 4).

**Table 4:** Procedural outcomes and adverse events

Characteristics	n/N (%)^1^
Post-LITT ECOG PS^2^	
0	19/30 (63%)
1	10/30 (33%)
3	1/30 (3.3%)
Post-LITT Decline in ECOG PS^2^	
No	24/30 (80%)
Yes	6/30 (20%)
Adverse events	
No	20/30 (67%)
Yes	10/30 (33%)
Transient neurological deficits	
No	22/30 (73%)
Yes	8/30 (27%)
Persisting neurological deficits	
No	27/30 (90%)
Yes	3/30 (10%)
Landriel-Ibañez Classification	
Ia	4/30 (13%)
Ib	4/30 (13%)
IIb	2/30 (7%)
> IIb	0/30 (0%)
Length of stay (days)	
1	21/30 (70%)
2	5/30 (17%)
4	1/30 (3.3%)
5	1/30 (3.3%)
7	2/30 (6.7%)

^1^n/total (%)

^2^ECOG Performance status

The median length of postoperative hospitalization was 1 day (IQR 1–2 days). Discharge occurred on postoperative day 1 for 21 patients (70%) and by day 2 for 26 patients (87%). Two patients (7%) remained hospitalized for 4–5 days, and two patients (7%) for 7 days.

### Survival outcomes

**Fig. 2:**
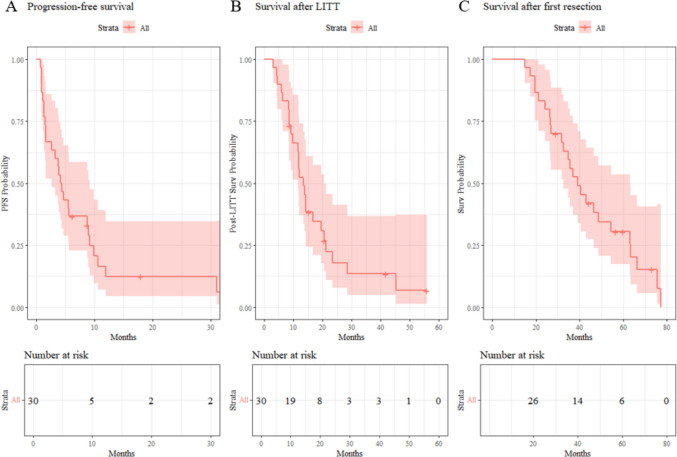
Progression-free survival, survival after LITT and survival after first resection. Kaplan–Meier survival analysis for the 30 patients treated with LITT for recurrent glioblastoma. (**A**) Progression-free survival (PFS), defined as the time from LITT to radiographic progression. (**B**) Survival after LITT, defined as the time from LITT to death or last follow-up. (**C**) Survival after first resection, defined as time from first surgery to death or last follow-up. All survival curves are accompanied by risk tables showing the number of patients at risk at each time point. Time is shown in months on the x-axis, and survival probability on the y-axis. PFS = progression-free survival; Surv = survival; LITT = laser interstitial thermal therapy

Median PFS was 4.3 months (95% CI 2.6–9.1) with PFS after 6 months in 37% (95% CI 0.23%−0.59%), and median overall survival from the date of LITT was 13.5 months (95% CI 11.6–20.5) (Table 5, Fig. 2). Median overall survival from primary resection was 39.2 months (95% CI 32.6–63.1).

**Table 5:** Survival outcomes

Characteristics	Value^1^
Alive at time of analysis	
No	25/30 (83%)
Yes	5/30 (17%)
Survival after LITT (months)	13.5 (11.6, 20.5)
Survival since first resection (months)	39.2 (32.6, 63.1)
Progression at time of analysis	
No	3/30 (10%)
Yes	27/30 (90%)
Progression free survival (months)	4.3 (2.6, 9.1)
Progression free survival at 6 months	37% (0.23%−0.59%)
Pattern of progression	
Local and distant	12/30 (40%)
Local	15/30 (50%)
No recurrence	3/30 (10%)

^1^n/total (%); Median (95% Confidence Interval)

By last follow-up, 27 of 30 patients (90%) had experienced progression. Local in 15 (50%) and distant in 12 (40%); three patients (10%) remained progression-free. Five patients (17%) were alive at analysis.

A subgroup analysis compared post-LITT survival in patients with biopsy-confirmed tumor (*n* = 18) versus those without biopsy (*n* = 11). The estimated survival probability at 12 months was 60% (95% CI 41%–88%) in the biopsy-confirmed group compared with 46% (95% CI 24%–87%) in the non-biopsy group. This difference was not statistically significant (log-rank p = 0.997).

## Discussion

This two-center study demonstrates that LITT is feasible and generally well tolerated, and that meaningful PFS and OS were observed following LITT in this selected cohort of patients with IDH-wildtype rGBM. Among the 30 patients included, the median survival after LITT was 13.5 months, with a PFS of 4.3 months, and durable tumor control for more than 6 months was observed in 37% of patients. Our survival outcomes are consistent with existing literature on LITT for rGBM. Recent meta-analyses have reported median post-LITT survival of approximately 12 months and progression-free survival of 4.6 months [15, 27]. The outcomes observed in our cohort fall within this expected range, supporting the use of LITT as a viable treatment modality.

To our knowledge, this is the first multi-institutional European series reporting outcomes of LITT for rGBM, thereby contributing real-world data to the existing literature [15]. A strength is the high rate of biopsy confirmation; 63 percent of patients underwent sampling and 95 percent of those specimens demonstrated viable tumor, compared with a reactive-change rate of approximately 13 percent in one of the largest published biopsied suspected tumor recurrence cohorts [7]. Despite potential differences in patient selection, lesion characteristics, and adjuvant therapy practices between continents, our post-LITT survival outcomes in a consecutive series with no patients excluded closely parallel those reported in North American series, supporting the generalizability of LITT across diverse practice settings.

However, several limitations reduce comparability with established treatment paradigms such as repeat surgical resection. **First**, the relatively low tumor volume and distant tumor location in our cohort limits direct comparison to craniotomy series, where tumors are typically larger and located in close relation to the resection cavity. **Second**, not all patients underwent biopsy prior to LITT due to initial technical concerns raised when combining biopsy and ablation in the same session. This raises the possibility of misclassification, as some presumed tumor recurrences may have represented radiation necrosis. That said, tumor cells were identified in 95% of biopsied patients, and survival outcomes did not differ significantly between those with biopsy-confirmed recurrence and those without. **Third**, this cohort demonstrated a notably long overall survival, with a median of 39.2 months and a median time from initial surgery to LITT of 18.1 months. This suggests selection bias toward patients with more favorable prognoses. Nevertheless, LITT may have contributed meaningfully to this extended survival by effectively controlling local tumor progression in selected patients.

Furthermore, it is also important to recognize that this case series reflects our early institutional experience with the technique. Patient selection, procedural planning and surgical technique evolved over the course of the study, which may impact generalizability when comparing to more established programs or larger series.

The overall rate of adverse events was 33%, primarily involving transient neurological deterioration. These adverse events are more plausibly attributed to the anatomical complexity and proximity of lesions to critical white matter tracts or deep brain nuclei, rather than inherent limitations of the LITT technique. LITT allows for precise and controlled thermal ablation with sharply defined margins, which helps to minimize injury to adjacent brain structures when favorable anatomy permits. Despite these adverse events, the procedure was generally well-tolerated, functional preservation in 80% and most patients (84%) were discharged home within 48 h.

Beyond cytoreduction, LITT may play a role in modulating the tumor microenvironment and may facilitate immunogenic cell death, priming the immune system for enhanced antitumor response [8, 22–24]. Furthermore, thermal ablation disrupts the blood–brain barrier reflected by a perilesional contrast-enhancing rim which may serve as a conduit for transferring antitumoral therapy across this barrier [9, 10, 13, 18, 20]. Preclinical and clinical studies are increasingly exploring combinations of LITT with systemic therapies, including immunotherapy and otherwise BBB-impermeable agents [3, 5, 22].

Despite encouraging retrospective data, including our own, a critical evidence gap remains. A randomized controlled trial comparing LITT and repeat craniotomy or oncological therapy alone for recurrent glioblastoma is needed. Such a trial should include standardized eligibility criteria, uniform postoperative therapies, and endpoints encompassing progression-free survival, overall survival, quality of life, cognition, neurological outcomes, and timing of systemic treatment initiation.

Until high-level evidence is available, LITT should be considered a viable, minimally invasive cytoreductive tool in the neuro-oncologic armamentarium. Particularly when conventional resection is rendered challenging due to anatomical or patient-specific factors. With continued refinement in patient selection and increased integration with adjuvant therapies, the role of LITT is likely to expand in the management of rGBM.

## Conclusions

In this multicenter Scandinavian cohort, LITT was demonstrated to be a feasible and generally well tolerated cytoreductive option for selected patients with rGBM. Procedural parameters, including low incidence of permanent morbidity and short postoperative hospitalization, are consistent with the minimally invasive nature of this technique. Survival outcomes observed in this series were comparable to those reported in published North American LITT cohorts, and functional status was preserved in most patients. While these findings suggest that LITT represent a viable treatment option in selected cases, its clinical value relative to other salvage strategies remains uncertain. Further evaluation in prospective studies, ideally with standardized patient selection, procedural techniques, and post-treatment management, is warranted to better define the role of LITT in the management of recurrent glioblastoma.

## Data Availability

No datasets were generated or analysed during the current study.

## Abbreviations

BBB: Blood–brain barrier
CI: Confidence interval
CRW: Cosman–Roberts–Wells
ECOG PS: Eastern Cooperative Oncology Group performance status
GBM: Glioblastoma
IQR: Interquartile range
IDH: Isocitrate dehydrogenase
LITT: Laser interstitial thermal therapy
MGMT: O6‑methylguanine‑DNA methyltransferase
MRI: Magnetic resonance imaging
OS: Overall survival
PFS: Progression‑free survival
rGBM: Recurrent glioblastoma
RANO: Response Assessment in Neuro‑Oncology
